# Gravitational and magnetic field variations synergize to cause subtle variations in the global transcriptional state of *Arabidopsis in vitro *callus cultures

**DOI:** 10.1186/1471-2164-13-105

**Published:** 2012-03-21

**Authors:** Ana I Manzano, Jack JWA van Loon, Peter CM Christianen, Juana M Gonzalez-Rubio, F Javier Medina, Raul Herranz

**Affiliations:** 1 Centro de Investigaciones Biológicas (CSIC), C/Ramiro de Maeztu 9, E-28040 Madrid, Spain; 2 Dutch Experiment Support Center, DESC at OCB-ACTA, Free University and Univ. of Amsterdam, Amsterdam, the Netherlands; 3 High Field Magnet Laboratory (HFML), Institute for Molecules and Materials, Radboud University Nijmegen, Nijmegen, The Netherlands; 4 Centro Nacional de Biotecnología (UAM-CSIC), Madrid, Spain

## Abstract

**Background:**

Biological systems respond to changes in both the Earth's magnetic and gravitational fields, but as experiments in space are expensive and infrequent, Earth-based simulation techniques are required. A high gradient magnetic field can be used to levitate biological material, thereby simulating microgravity and can also create environments with a reduced or an enhanced level of gravity (*g*), although special attention should be paid to the possible effects of the magnetic field (*B*) itself.

**Results:**

Using diamagnetic levitation, we exposed *Arabidopsis thaliana in vitro *callus cultures to five environments with different levels of effective gravity and magnetic field strengths. The environments included levitation, i.e. simulated μg* (close to 0 g* at *B *= 10.1 T), intermediate g* (0.1 g* at *B *= 14.7 T) and enhanced gravity levels (1.9 g* at *B *= 14.7 T and 2 g* at *B *= 10.1 T) plus an internal 1 g* control (*B *= 16.5 T). The asterisk denotes the presence of the background magnetic field, as opposed to the effective gravity environments in the absence of an applied magnetic field, created using a Random Position Machine (simulated μ*g*) and a Large Diameter Centrifuge (2 *g*).

Microarray analysis indicates that changes in the overall gene expression of cultured cells exposed to these unusual environments barely reach significance using an FDR algorithm. However, it was found that gravitational and magnetic fields produce synergistic variations in the steady state of the transcriptional profile of plants. Transcriptomic results confirm that high gradient magnetic fields (i.e. to create μg* and 2 g* conditions) have a significant effect, mainly on structural, abiotic stress genes and secondary metabolism genes, but these subtle gravitational effects are only observable using clustering methodologies.

**Conclusions:**

A detailed microarray dataset analysis, based on clustering of similarly expressed genes (GEDI software), can detect underlying global-scale responses, which cannot be detected by means of individual gene expression techniques using raw or corrected p values (FDR). A subtle, but consistent, genome-scale response to hypogravity environments was found, which was opposite to the response in a hypergravity environment.

## Background

Since the beginning of life on Earth, organisms have lived under the influence of the Earth's physical parameters including its almost constant gravitational and magnetic fields. Therefore, evolution has had to provide a number of different solutions to meet the mechanical challenge of supporting the weight of a living organism [1]. In general, the influence of gravity on the physiology of an organism increases with its mass, although for organisms living in water, the effect of gravity is to some extent mitigated by buoyancy. In plants, gravity has an important effect on the development of small seedlings via the sedimentation of heavy components (statoliths), but gravitational effects in non-specialized cells have also been reported [2,3]. The reduced gravity on the surfaces of Mars (0.38 *g*) and the Moon (0.17 *g*) may significantly affect not only astronauts manning the first space colonies, but also the development of plants, which would be an essential part of life support systems. It is also possible that zero- and reduced-gravity might have unexpected effects on the behaviour of bacteria, viruses and other micro-organisms, either directly or through the effect of reduced gravity on the environment, e.g. through a modified convection in gases and fluids [4]. The natural magnetic field strength at the surface of the Earth varies from 30 to 60 μT [5], but magnetic fields of up to 12 T are being used regularly in diagnostic non-invasive techniques, such as magnetic resonance imaging [6,7], without any apparent long-lasting effects on cells. Nevertheless, there have been reports that high magnetic fields affect bacteria [8], plants [9-11], mammals [12], and flies [13]. The exposure of *Arabidopsis *seedlings to magnetic fields in excess of about 15 T for 6.5 h has been linked with 2.5 fold changes in the expression levels of 114 genes [14].

To study the effect of an environment with altered gravitational forces, we have to place samples in orbit by means of space flights, sounding rockets, or use simulation facilities on the ground. Mechanical facilities such as 2D-clinostats or random positioning machines (RPMs), and for enhancement, centrifuges like the Large Diameter Centrifuge (LDC) [15-18], have been used for decades in well-equipped laboratories to average out the gravitational force. A critical evaluation and quantification of the exact physical effects involved in these simulators is, however, often missing. Nevertheless, such an evaluation would help to answer the question as to whether using such machines creates either a stimulus-free environment with respect to gravity (= simulated weightlessness) or an omnilateral gravistimulation with strong mechanical disturbances. An alternative and relatively new approach to study the response of organisms to changes in gravity is the use of diamagnetic levitation [19-24]. Since diamagnetic material is repelled by magnetic fields, when a diamagnetic object is positioned in a magnetic field gradient it experiences a magnetic force away from regions of high field. The size of the force is proportional to the product of the field strength (*B*) and the field gradient (the spatial derivative of the field *B'*). When *B *times *B' *is strong enough, this magnetic force can be used to counterbalance the gravitational force, leading to the stable levitation of a large variety of materials such as water and other fluids. Since the bulk of living organisms is composed of diamagnetic material, mostly water, organisms can be magnetically levitated, requiring *BB' *to be equal to about 1400 T^2^/m, which in commonly used magnets occurs at a field strength of about 16 T. Such conditions can now be readily produced in a dozen facilities around the world.

In this paper, we have studied the effect on the overall transcriptional state of *Arabidopsis thaliana *semi-solid cell cultures (callus) exposed to an environment of altered gravitational and magnetic forces for 200 min. This biological system, composed of undifferentiated proliferating cells, was chosen in the context of our previous investigations on the effects of gravity alteration on cell growth and proliferation [25,26]. Our first goal in this analysis was to test what kind of facility would be most suitable and reliable as an altered gravity simulator. Previous investigations used different experimental settings for each facility, limited their analysis to a particular collection of genes or used a low number of seedlings per sample compromising statistical outcomes [3,14,27,28]. The present study is the first systematic multi-facility, high-throughput, environmentally controlled collection of experiments that has been performed with the same set-up, almost simultaneously in two mechanical facilities (RPM for μg and LDC for 2 *g*) and a magnet-based facility using five different effective gravity (g*) conditions (from μg* to 2 g*). This was to allow sustainable inter-experiment comparisons of the results and a pooled analysis with multiple inner controls at similar magnetic fields. During our research, we detected a synergic effect when more than one environmental parameter was affecting the samples (gravitational and magnetic fields) that could be linked to the collapse of cellular strategies to support environmental stresses already observed in space samples [29].

## Methods

### Levitation magnet description and set up

**Figure 1:**
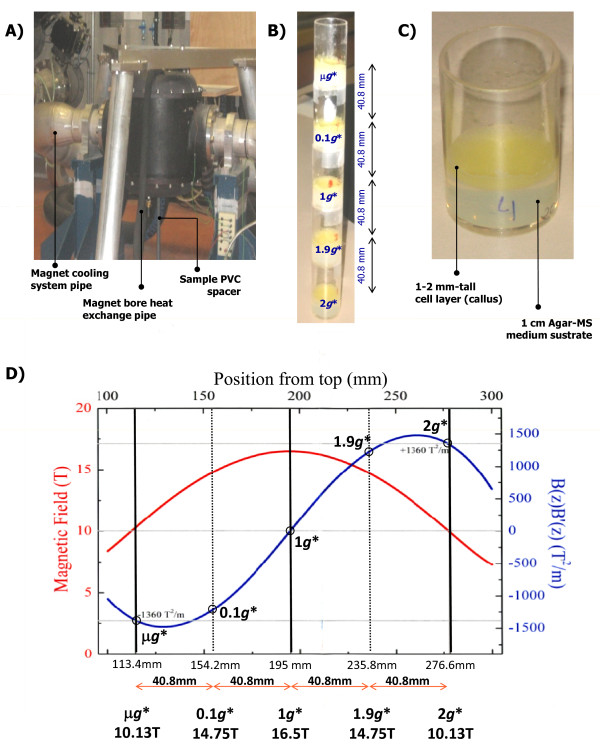
**Magnetic levitation experiment set up**. **A) **Photo of the water-cooled duplex-Bitter magnet located at HFML with our samples placed inside (not visible). The samples are positioned inside the magnet bore. The temperature is controlled by a double-walled metal tube connected to a 22°C water bath. A PVC spacer is used to place the stack of samples in the correct position. **B) **The samples are contained in 40.8 mm high tubes placed on top of each other at five effective g* levels. The spacing between the samples was 40.8 mm and all samples were in the dark before and during the experiment (no light reached the magnet bore). **C) **Closer view of a sample tube. Callus cell culture is grown in a 1-2 mm layer to ensure a similar force throughout the whole biological sample. **D) **Profile of the magnetic field strength (*B*) and the effective gravity (g*) as a function of position inside the magnet. The samples were placed symmetrically in relation to the centre of the bore (195 mm above the top) indicated in the graph by vertical lines (straight lines for μg*, 1 g* and 2 g* and dotted lines for intermediate 0.1 g* and 1.9 g*). The red curve shows the magnetic field strength as a function of the vertical position (*z*) in the magnet. The blue curve indicates the product of the field strength *B(z) *and the field gradient *(B' (z) = dB/dz)*, which is the derivative of the field strength with respect to the vertical position. The corresponding value of the effective gravity is equal to *g(1 + B(z) B' (z)/1360)*, so a magnetic force of -1360 T^2^/m is able to levitate water.

We exposed samples to five different conditions (g* and *B *fields) within a magnet located in the High Field Magnet Laboratory (HFML) at the Radboud University Nijmegen, The Netherlands [30,31]. The asterisk in g* indicates the presence of the background magnetic field, whose spatial profile is shown in Figure 1. In the centre of the magnet bore (positioned 195 mm from the top of the magnet) the magnetic field strength is maximal (16.5 T in our case). Here the magnetic field gradient is zero and, therefore, the magnetic force is also zero, leaving gravity unaffected; this is the 1 g* point (Figure 1). At a distance of 81.6 mm above the centre position within the solenoid (113 mm from the top), the diamagnetic force on water balances the force of gravity (at *B *= 10.1 T and *BB' *= 1360 T^2^/m), leading to stable levitation; this is the μg* point (zero gravity 0 g* is only reached in a single point). An intermediate sample was placed at 40.8 mm above the centre, where the calculated residual *g *force is 0.1 g* and the magnetic field 14.7 T. Below the centre of the magnet the magnetic force acts in the same direction as gravity, leading to enhanced gravity levels. Two samples were placed in positions with 1.9 g* (*B *= 14.7 T) and 2 g* (*B *= 10.1 T) forces (Figure 1 and Additional file 1: Figure S1). In order to discriminate between the effects due to the high magnetic field and field gradients and those due to the altered gravity, we carried out five 200 min simultaneous experiments exposing the samples at these 5 positions at 22°C. External controls, at 1 *g*, were kept at RT (22°C ± 0.1°C, 1 *g *ground gravity) away from the magnet (the magnetic field is negligible beyond the 4 metre security radius). The temperature in the magnet was controlled using a double-walled metallic holder tube with a thermostated water bath. The availability of the magnet and the cost of the experiments restricted the experimental time to 200 min.

### Mechanically altered gravity device descriptions and set up

We exposed samples to simulated microgravity in a Random Positioning Machine (μ*g*-RPM, real random mode) and to hypergravity in the Large Diameter Centrifuge (2 *g*-LDC) located in the European Space Research & Technology Centre (ESA-ESTEC) in Noordwijk, The Netherlands [17,32]. The RPM run was performed using a real random mode with a maximum rotational speed of 60 rpm and a rotational angle of 6°. In order to differentiate between the effects due to the mechanical load applied during 3D-rotation and centrifugation, we carried out the experiments simultaneously in the RPM and LDC, along with two external controls (on the RPM scaffold and at the central gondola of the LDC, see Additional file 1: Figure S1) for 200 min at RT (registered as 22°C ± 0.5°C). It should be noted that the temperature of the LDC room can be controlled, but that the internal temperature of the gondola is not controlled (the maximum recorded difference in temperature was 1°C between the experimental gondola at 2 *g *and the central gondola at 1 *g *which is located above the centrifuge engine and can become overheated during operation).

### Design and hardware of the biological experiments

Callus semi-solid cultures of *Arabidopsis thaliana *were prepared from MM2d suspension cultures as described in previous literature [33]. The best cultivation conditions for the MM2d culture are without light, so all processing and experiments were done in the dark (aluminium coverage of the samples during transportation and the LDC/RPM experiments, a PVC cap was used in the magnet experiments to prevent ambient light from entering the magnet bore, Figure 1A). For the magnet experiments the samples were prepared in 40.8 mm high by 25 mm diameter tubes that were taped head-to-tail to form a column divided into 5 levels with an opaque non-magnetic cap at the top (Figure 1B). Three identical magnet runs were performed. For the single run LDC/RPM experiment the samples were prepared in two regular 90 mm diameter Petri dishes (Additional file 1: Figure S1). The biological material was grown as a 1-2 mm thick layer on the surface of a 1 cm layer of 1% agar with MS medium. Due to this limited thickness, variations in the magnetic field and of the effective g-force are minimized (Figure 1C). For all devices and conditions, the suspension cultures were strewn on the agar surface one week before the start of the experiment and grown at 22°C to allow the callus to reach maximum density (1-2 mm thick layer). The samples were preserved immediately after treatment (less than 2 min) by quick freezing in liquid nitrogen and subsequent dry ice storage.

### RNA extraction and labelling protocol description

Total RNA was extracted from the frozen callus using TRIzol reagent (Invitrogen, 15596-026) according to the manufacturer's instructions and purified with an RNeasy mini kit (Qiagen, 74104). RNA quantity and quality was verified firstly by gel electrophoresis and Nanodrop spectrophotometry and later by using a Bioanalyzer. Two μg of the total RNA were amplified via the aRNA MessageAmp II kit (Ambion, 1751). Then, 7.5 μg of aminoallyl-labelled aRNA (experiment/control) were rinsed in 0.1 M Na_2_CO_3 _(pH 9.0) and labelled with Cy3/Hyper5 Mono NHS Ester (CyTMDye Post-labelling Reactive Dye Pack, Amersham). The samples were purified following the manufacturer's instructions for Megaclear TM (Ambion).

### Array protocol and sample pooling

The whole genome transcriptional profile was determined by three independent hybridizations of an experimental RNA and a control RNA sample for each condition using two color AGILENT microarrays of *Arabidopsis thaliana *(Agilent *Arabidopsis *Oligo Microarrays v4 slides catalogue number 021169 from Agilent Technologies, Inc. Headquarters (Santa Clara, United States) http://www.home.agilent.com/agilent/) hybridised in accordance with the manufacturer's specifications. The microarray selected probe sets signal was also determined by real time qPCR for validation ([34] Additional file 1: Figure S2). Each RNA sample pair (experimental and control) is a biological replica since it was collected from independent magnet experiments and from different *Arabidopsis *callus plates in the case of the RPM/LDC experiments. In the case of the 1.9 g* experimental condition only two samples of enough quality were obtained.

### Hybridization and scanning protocol description

For each hybridization, 825 ng of each sample (experiment/control) were mixed with 11 μl of 10 × blocking agent in a final volume of 52.8 μl of RNase-free water. Labelled aRNA was fragmented by adding 2.2 μl of 25 × Fragmentation buffer (Agilent) and incubating at 60°C for 30 min. The reaction was stopped with 55 μl of 2 × GEx hybridation buffer HI-RPM (Agilent) and applied to the slide. The slides were then incubated at 65°C for 17 h in hybridization chambers. After incubation, the slides were washed with wash buffers (Agilent) for 1 min. The slides were dried by centrifugation at 563 *g *for 1 min. Images from Cy3 and Cy5 channels were equilibrated and captured with a GenePix 4000B (Axon) and spots were converted into numerical data using GenPix software (Axon).

### Bioinformatics analysis based on Agilent microarray data

The microarray dataset has been submitted to the *Gene Expression Omnibus (GEO) *database with the accession number [GEO:GSE29787]. The gpr files were primary modified, to remove internal controls (EQCS, according to the manufacturer's recommendations) to carry out the analysis. The local background was corrected by the normexp method with an offset of 50. The background corrected intensities were transformed to log scale (base 2) and normalized by loess for each array [35]. Finally, to have similar intensity distributions across all arrays, the loess-normalized-intensity values were quantile normalized [36].

### Microarray data analysis

After data processing each probe was tested over replicates for changes in expression between different conditions using an empirical Bayes moderated t statistic, i.e. Limma [37] and/or Rank Products [38]. To control the false discovery rate (FDR), Limma and Rankprod p values were corrected using the method of Benjamini and Hochberg [39]. FIESTA viewer (bioinfogp.cnb.csic.es/tools/FIESTA) was used to visualize all microarray results and to evaluate the numerical thresholds applied for selecting differentially expressed genes [40]. Probe sets list were filtered using limma &/or rankprod-FDR p values depending on the stringency of the statistical tests required for each analysis from the FIESTA viewer interface. From the approximate 44 k probe set list included in the Agilent microarrays, a list of 2470 probe sets were detected to show a change in at least one condition with the lower stringency test. Gene lists were obtained by choosing the probe set with the higher signal variation when more than one probe set is available for the same gene.

### Gene ontology and whole genome GEDI analysis

Gene ontology was analyzed in the selected probe set lists by BINGO 2.3 analysis using default software settings [41]. BiNGO currently provides a statistical test for assessing the enrichment of a GO term in a set of genes (using a hypergeometric test p value with the Benjamini and Hochberg FDR correction) that are up- or down-regulated in a microarray experiment, and pasted in a text input box. A global and integrative analysis using "gene expression dynamics inspector" (GEDI) self-organizing maps, was performed using the above indicated software v2.1 [42]. Using transformed and corrected signal log2ratios data, we identified 17,419 probe sets that show signal log2ratio changes > 0.5 or < -0.5 relative to the 1 g control in at least one of the experimental conditions. Mosaics of 20 × 16 grid size (average of 54 probe sets/tile) were obtained using the self-organizing maps algorithm and standard settings of the software [42] using the signal log2ratio of the selected probe sets. The average signal log2ratio for each tile or cluster of probe sets was calculated and displayed in panels for any of the experimental conditions analyzed and also for virtual condition panels (average signal log2ratio of 1 g* internal controls was subtracted from each magnet condition average signal log2ratio trying to remove high magnetic field effects from high gradient magnetic fields that modify net weight).

## Results

### An exposure of 200 min to intense magnetic fields alters the microarray-based transcriptional profile in *Arabidopsis *callus

**Figure 2:**
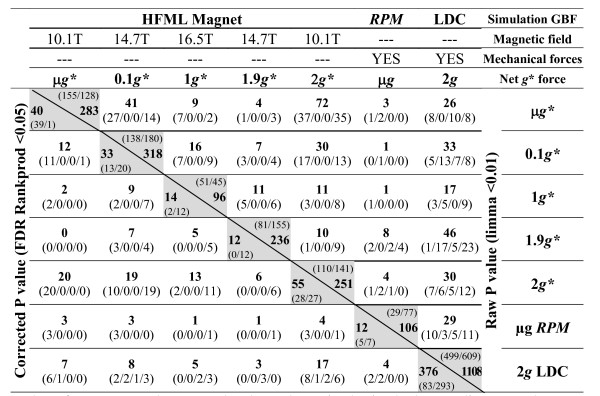
Number of genes showing expression changes (up- or down- regulation) under different effective gravity (g*) and magnetical/mechanical conditions. Number of genes up- or down- regulated was determined using both a raw limma p value < 0.01 (above the diagonal line) and a corrected FDR Rankprod p value < 0.05 (below the diagonal line) by FIESTA viewer v.1.0. Total number of genes up- or down-regulated is shown in bold. In diagonal (grey shaded) we show the number of gene expression changes in each condition (up-regulated/down-regulated genes between brackets). Other cells show the number of genes in common between two conditions (up-regulated in both/up-regulated in the column condition & down-regulated in the row condition/down-regulated in the column condition & up-regulated in the row condition/down-regulated in both conditions between brackets). This information has been extracted from Additional files 2 and 3 tables containing quantitative expression data for these probe sets and the list of common genes in more than one condition using limma p value filter and also FDR (RankProd) filter.

**Figure 3:**
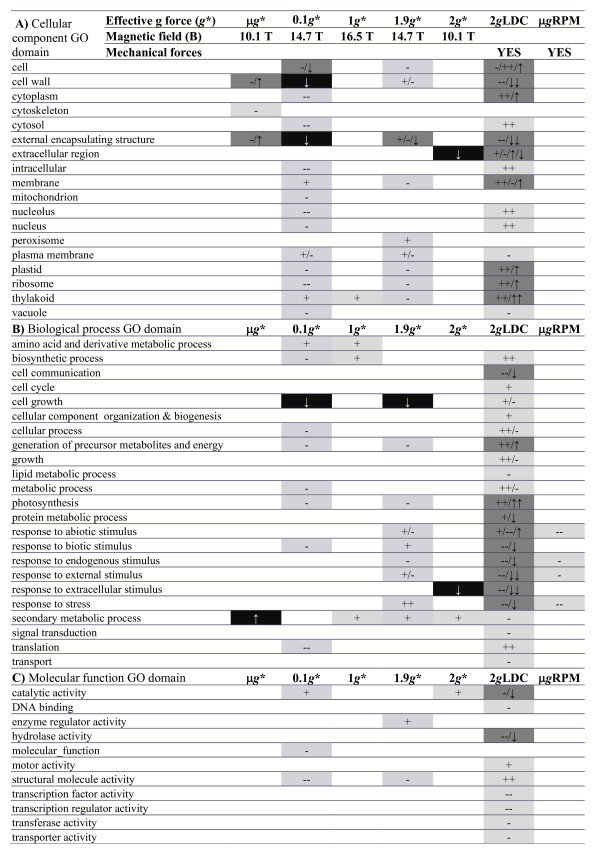
**Analysis of enriched GO groups in genes showing significant altered expression in the different experimental conditions by GO domains**. **A) **GO Cellular component, **B) **GO Biological process &**C) **GO Molecular function. We have use the symbols +/- when the list of genes used as input for the BINGO GO tool were filtered using limma p value and ↑/↓ when the lists were filtered using the FDR-rankprod p value, analyzing up- (+/↑) and down-regulated (-/↓) gene lists separately. The following GO groups are significantly overrepresented in each sample as determined using BINGO 2.3 with default settings (one symbol indicate a p < 0.001 and two symbols a p < 0.0005 using a hypergeometric test with BH-FDR algorithm). We use shading to indicate if this GO term is overrepresented using limma p value input list (soft shaded) only, both lists (dark shaded) or FDR rankprod p value list only (black shaded, white letters).

Figure 2 reflects the number of genes whose signal level changes in the different altered gravity/magnetic field environments, compared with the 1 *g *controls outside the simulators with a raw limma p value < 0.01 (above the diagonal) and a FDR corrected RankProd p value < 0.05 (below the diagonal). To determine the effects of the magnetic field alone we needed to pay attention to the effects on the internal 1 g* control and common genes in other positions. Using a raw limma p value, after 200 min in the magnet 96 genes showed significant alterations in the 1 g* position (in which *B *= 16.5 T without changing the effective *g *force. See Additional file 2: Table S1 for quantitative gene expression data) equally distributed between up- and down-regulated genes. On studying the gene ontologies (GOs) affected in this group of genes (Figure 3) we found significant enrichment in some biosynthetic and metabolic processes including thylakoid-related genes, all of them over-expressed. Comparison between different positions in the magnet (values out of the main diagonal in Figure 2) offers a low number of common genes (below 5%) except for the μg* vs 0.1 g* and the μg* vs 2 g* positions. Importantly, all miss-regulated genes in the magnet samples behaved similarly under both conditions (second and third values between brackets are 0) suggesting that these gene expression variations are related more with the high magnetic field (> 10 T in any position) than with the differential net force between them. Analysis of these common genes suggests a general stress response involving multiple enzyme activities that can be related to the presence of the high magnetic field (tranferases and peroxidases, see Additional file 3: Table S2). Using stringent statistical tests (RankProd FDR p value < 0.05) we obtained a lower number of affected genes (14), but equally distributed between up- and down-regulated genes, and enriched GO groups (Figure 3).

### Magnetic field and effective gravity alteration promote a synergic repression of the microarray-based transcriptional profile

Samples at different positions in the magnet experience different levels of effective gravitational force and magnetic field strength. In the two hypogravity (μg* and 0.1 g*) and the two hypergravity (1.9 g* and 2 g*) positions we observed a 3 fold increase in the number of probe sets that showed signal variations compared with the 1 g* position (Figure 2). Three points should be stressed here regarding our raw limma analysis results. First, the number of genes altered in the hypogravity positions is higher than those altered in the hypergravity positions (283 and 318 for hypogravity versus 236 and 251 for hypergravity). Second, the number of up-regulated genes is higher in environments with high gravitational deviation (138 in 0.1 g* to 155 in 0 g* for hypogravity and 81 in 1.9 g* to 110 in 2 g* for hypergravity). Finally, the amount of down-regulated genes behaves oppositely to the up-regulated genes, being higher in the intermediate positions (0.1 g* and 1.9 g*) where the *B *field is higher and more similar to the one in the centre of the bore (14.7 T versus 16.5 T in the centre of the bore and 10.1 T in the μg*/2 g* positions, Figure 2). It is important to remember here, that as illustrated in Figure 1D, the magnetic field variation is even steeper than the gravitational variation between them. Using FDR corrected analysis the results are less evident but consistent with the low stringency analysis.

In terms of gene ontology, although enrichment was affected by the magnetic and gravitational field altered environments (Figure 3), we detected that few categories were affected in the μg* and 2 g* positions. GO related with the simulated microgravity condition include cell wall, cytoskeleton and encapsulating structures, all of them appearing in down-regulated lists using p value limma but in the up-regulated list when using RankProd FDR values. In the 2 g* position only catalytic activity and secondary metabolism genes are enriched, being up-regulated. Finally, we turn to the intermediate positions, in which the effective gravity alteration is still remarkable (0.1 g*/1.9 g*) but with a magnetic field that is 50% stronger than in the μg*/2 g* position and almost similar to that in the 1 g* position. Here, despite the fact that the total number of genes affected is quite similar, the number of GO groups that appear enriched is much larger, affecting a long list of cellular functions when using p value limma results.

### Magnetic and mechanical simulators alter the microarray-based transcriptional profile in different ways

Using mechanical simulators we obtained a different picture. Using an RPM to compensate gravity we obtained 106 genes that showed a significant modification in the signal levels of the microarray hybridizations (raw limma p value), but only a quarter of them exhibited over-expression (Figure 2). In terms of GO we observed that the 77 down-regulated genes were enriched in response to stress and abiotic stimulus genes (Figure 3). The results obtained with double gravity (2 *g*-LDC) were surprising. The number of genes affected in this 2 *g *environment was 6 times more than in the 2 g* environment (even in the absence of a magnetic field, Figure 2). With regard to GO, almost all groups identified in the previous experiments were also affected in this group, resembling the effect observed in the intermediate 0.1 g* and 1.9 g* positions (Figure 3). Remarkably, both raw limma and FDR RankProd algorithms offer quite similar results in the case of LDC conditions, confirming that the findings with both algorithms are similar when the list of genes is long enough for this GO analysis.

### Changes in environmental parameters contribute to a fine-tuning effect on the microarray-based transcriptional profile

**Figure 4:**
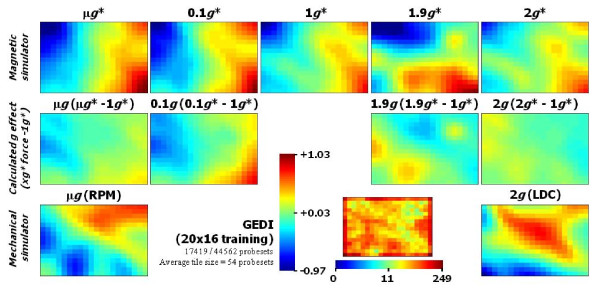
GEDI whole-genome transcriptional status of the samples exposed to different g* and B fields. A 20 × 16 clustering analysis is shown based on the five magnetic experimental conditions (first row panels) and the two mechanical experimental conditions (third row panels) versus the external 1 *g *control. The panels in the second row have been calculated by extracting the 1 g* panel values (only magnetic effect) from the g* panels immediately above. The vertical colour scale bar indicates the average log2ratio levels of each cluster in the conditions compared to the parallel 1 *g *control (first and third row) or versus the 1 g* control (second row). The average signal in experimental conditions is slightly higher than the 1 *g *control (log2ratio equal to 0.03 in the centre of the scale bar) suggesting overall up-regulation. Double up-regulated clusters (with an average log2ratio > 1.03) are saturated to red and those half down-regulated (average log2ratio < -0.97) are saturated to blue. Clusters in between follow a continuous colour scale as indicated. The centre panel indicates the number of probe sets included in each cluster (20 × 16 clusters with an average size of 54 probe sets per pixel) following its own horizontal scale at the bottom. Source GEDI files are available as Additional file 4.

Although the changes mentioned above affected a relatively low number of genes, we wanted to evaluate the overall outcome of the transcriptional profile of *Arabidopsis *callus exposed to these anomalous environments. We analysed the microarray data with the "Gene Expression Dynamics Inspector" (GEDI) program [42]. GEDI is a "Self Organizing Map" based software that allows the visualization of whole genome expression patterns in mosaics of *n *× *m *tiles. Each tile corresponds to a cluster of genes that share a similar gene expression pattern across conditions (centroid). Different colours reflect the expression intensity of a centroid in each condition (in our case the average ratio of intensities compared to 1 *g *controls). Additionally, GEDI places similar centroids close to each other in the mosaic, creating an image of the transcriptome and allowing its analysis as an entity by simple visualization and through different conditions. For this analysis we avoided filtering the data with any p value that could hide information, which meant normalizing the expression data and removing probe sets without at least a 0.5 fold change in any condition. Accordingly, 17419 of 44562 probe-sets were finally used for the GEDI analysis. They were placed in 20 × 16 mosaics with an average of 54 genes per centroid. Figure 4, shows examples of similar analyses [29,43]. When comparing the transcriptional status panels with the magnetic simulator conditions (versus parallel external 1 *g *control) we observed similar but not identical patterns related to the high magnetic fields (10.1 to 16.5 Tesla, Figure 4 first row). In order to minimize the magnetic field effects on the altered gravity panels we subtracted the 1 g* panel signal ratios from the altered gravity panels (xg*-1 g* → xg, Figure 4 second row). The panels obtained after this simple operation corroborated two ideas. First, the effect of the magnetic field is greater than the effect of altered gravity in this context. Second, the μ*g*/0.1 *g *and 1.9 *g*/2 *g *panels are very similar to each other, but partially opposite to the hypogravity and hypergravity panels (blue repressed areas are substituted by yellow/red areas).

Finally, to compare magnetically and mechanically induced micro and hypergravity we included the panels of the samples obtained from the RPM and LDC experiments (Figure 4, third row). The resulting comparison between μg (RPM) and 2 *g *(LDC) offers an ambiguous picture, since the patterns seem to be unrelated, neither similar nor opposite. However, an inverted correlation between μg (RPM) and 1.9 *g *panels is very clear (two blue areas in microgravity are yellow/red in hypergravity). The correlation with 2 *g *(LDC) is probably obscured because the large number of genes that show variations in the LDC samples hinder the observation of the background transcriptional status. Nevertheless, the red diagonal spot in the 2 g panel may be related to genes up-regulated by the mechanical stress observed in a less clear way in the other mechanical simulator (RPM).

## Discussion

Exposure of *Arabidopsis *callus cultures to magnetically altered environments produces significant changes in the gene expression profiles. We have found 96 gene expression variations in a 200 min-long experiment in a 16.5 T magnetic field (1 g* position), very similar to the 114 genes with altered expression in a 6.5 h-long experiment exposing *Arabidopsis *plants to a homogeneous 21 T magnetic field [14]. It should be noted that we evaluated up to 44000 probe sets and defined a significant and quantitative response by a limma p value < 0.01 and without minimal fold change requirements. In contrast, the approach of Paul et al. [14] consisted of evaluating 8000 genes choosing a 2.5 fold change requirement. We validated the microarray expression values of some probe sets of particular interest for our research by qPCR. The expression ratios are consistent but slightly higher than those determined by microarray analysis [34], but not enough to reach the 2.5 fold values established by Paul et al. [14]. Those values were only found in some cases for our samples (in 12 probe sets in the 1 g* position, for instance as shown in Additional file 2: Table S1). In fact, our results are consistent with the weaker effects observed at 15 T as compared to the main study at 21 T [14].

The main finding reported here is the synergic effect of the simultaneous modification of the effective gravitational and magnetic fields. Both the number of altered genes and the GO groups affected increased when we applied a magnetic field gradient, thereby changing the effective gravity, especially when we maximized the variation of both environmental parameters (0.1 g* and 14.7 T). We can attribute this synergic effect to the concerted action of two environmental parameter alterations acting at the same time on a cell that is trying to fit its transcriptome into a new habitat. Similar fine-tuning effects of the transcriptome have also been observed in a very different organism, *Drosophila*, when exposed to altered gravity in suboptimal environmental conditions [29]. Some of the enriched GO groups in our magnetically exposed samples (e.g. induction of stress related genes and depression of cell wall metabolism) were already detected using constant magnetic fields [14] but other affected GO groups were different in each position in the magnet. It must be stressed here that it is possible that high magnetic field gradients and homogeneous magnetic fields can affect different biological processes (ponderomotive force vs. ion transport), so finding different GO groups would not be unexpected [44]. Magnetic fields (1 g* positions) affect biosynthetic processes, secondary metabolism and thylakoids, all of them related with ion fluxes; this has also been linked with typical stress responses in plants undergoing centrifugation [28]. In fact, it is not surprising that thylakoid related gene expression patterns are affected, since it is well known that these organelles align even with a homogeneous externally applied magnetic field as small as 1 Tesla [45]. In the position with maximal variation of the gravitational force (μg*) we appreciated GO group structural alterations in cell walls, cytoskeletons and external encapsulating structures, as well as a susceptibility to structures being magnetically reoriented in the cell. Intermediate positions showed an increase in the number of affected genes that could be assigned to particular functions in the cell (over-represented GO groups) thus supporting the synergy of stress theories. Some of the GO groups included in Figure 3 (e.g. secondary metabolism, cell wall component biosynthesis and rearrangement) have also been associated with stress/defence responses in short-term experiments exposing *Arabidopsis *to hypergravity or microgravity simulation [27,28]. In fact, other authors have discussed that some genes related to reductase activities can also be involved in the differential growth and properties of the cell wall under hypergravity [46]. Furthermore, these genes are also involved in the cellular pathways of environmental stimuli response, so their involvement in the response to magnetic or gravitational fields should be expected. The number of changes in gene expression obtained in the RPM experiment was comparable to the results found at the μg* position in the magnet, but the affected GO groups were not similar. Abiotic stress GO groups were very clearly affected and repressed in the case of RPM, while in the case of μg* the response was connected with structures in the cell. In fact, the response was more in line with the one observed in the 0.1 g* and 1.9 g* positions: possibly due to the sum of environmental factors causing the higher responsiveness of this experiment. Previous short-term experiments with *Arabidopsis *callus cultures suggested that RPM simulated microgravity is more comparable, in terms of gene expression studies, to magnetic levitation than to 2D-clinorotation [3]. In fact, clinorotation was more related to hypergravity (centrifuge) in the same experiment. We have to bear in mind that not only the magnetic field but also the mechanical forces that appear during the operation of these facilities can affect gene expression. Another explanation for the unusual 2 g/2 g* comparison relies on the observation that the effects of magnetic fields and microgravity are additive in the μg* position, but could be subtractive in the 2 g* position resulting in the magnetic field effect (similar to the μg* one) hiding the inverted-gravitational effect. This is a possible explanation that might be enhanced further by the LDC controlled environmental conditions or by vibration factors, that have affected the three levels of results presented here (Figure 2, Figure 3 and Figure 4).

From the paragraphs above and particularly from Figure 3 (GO enrichment analysis) an incorrect observation could be made that the overall transcriptional profile in the five magnet positions is very different. Although individual gene expression variations affect several GO domains, they only affect a few genes (less than 5% of the transcriptome). We found that less than 10% of these genes showed a significant variation in expression, even using the permissive raw limma p < 0,01) in more than one magnet position (Figure 2). In these cases the change was always in the same way, up- or down-regulating the gene expression. This suggests that a small number of common magnetically affected genes (less than 0.5% of the proteome) are being altered in all magnet positions, but that the major part of the genome is out of the statistically significant threshold. To clarify the situation we analysed the entire genome in one go with a GEDI analysis to evaluate the overall transcriptome status, and it proved to be quite similar in the five magnet samples (Figure 4 row 1), especially if we compare the hypogravity and hypergravity panels. So we have detected on the one hand, an overall transcriptome response that it is linked with a high magnetic field, and on the other hand a subtle response based on small variations or fine-tuning changes related to the gravitational effective force. If we focus on the microgravity panels we can see that the 0.1 g* effect is similar to the μg* effect but synergized by the increased magnetic field. Curiously, 0.1 g* hypogravity can be above the residual gravity threshold detectable by the statoliths in plants, even 10^-3 ^*g *effects are observable [47], but our culture cells did not contain statoliths so we must bear in mind that we are detecting a response independent of this mechanism. This is clearly observable if we subtract the 1 g* effect from the other g* conditions (Figure 4 row 2), both for hypo- and hyper-gravity samples. The comparison with mechanical simulators is more difficult due to the possible inertial effects and temperature perturbations as reflected in the hard to compare RPM and LDC transcriptome panels. These environmental interferences are very important in microarray-based ecology research [48], so performing experiments simultaneously (as in the magnet) is much appreciated. Nevertheless, the RPM panel resembles an inverted version of the 1.9 g*-1 g* panel (synergized hypergravity panel) suggesting that the fine-tuning factor has the opposite effect in hypergravity and hypogravity conditions. Additional file 1: Table S1 includes the GO analysis of these repressed or induced probe sets (blue or red areas), suggesting that genes involved in biological processes across the cell, specifically abiotic stress genes, behave oppositely to micro and hypergravity as in other model systems [29].

## Conclusions

Summarizing, we conclude that biological systems can respond to environments featuring altered physical forces, such as gravity or magnetic fields, by adapting the transcriptional status of their whole genome in a subtle but regulated way. Since this involves adapting to a change in an environmental parameter that has remained constant since the evolution of life on Earth began, it should be complex enough to depend on only a small number of key genes or pathways. Looking for particular master genes has misdirected this research for some time, but new high-throughput molecular tools are offering a new biological perspective. Investigation of purely microgravity effects should be performed in space, but mechanical and magnetic simulators could be used to study similar phenomena if we are able to distinguish the mechanical/magnetic effects from the gravitational effects in our systems. The magnet is the only available facility up to now, that allows us to simulate, at the same time and in the same stable environment, low gravity like that on Mars or the Moon and a hypergravity environment on the surface of the Earth. This is especially useful since we have found that transcriptional experiments are quite sensitive to small variations in environmental conditions. Consequently, magnetic levitation can be an alternative to other ground based methodologies, allowing testing of the biological effects of altered gravitational forces in an unusual environment.

## Competing interests

The authors declare that they have no competing interests.

## Authors' contributions

AIM supported by RH carried out on-site RPM/LDC/magnet samples processing, molecular genetic studies and the data analyses. JvL and PCMC carried out on-site set up and previous calibrations of the ground based facilities and collaborated in the optimization of set up design. JMGR perform the microarray hybridization, scanning and data analysis processing and submission to GEO database. FJM conceived the study and helped to draft the manuscript. RH drafted the manuscript and coordinated the study conception, design and performance. All authors read and approved the final manuscript.

## Supplementary Material

Additional file 1 **Supplementary online material including additional figures (S1 & S2) and Table S1 has been uploaded as a pdf file**. Click here for file

Additional file 2 Table S1. Quantitative expression of probe sets (fold change) showing induction or repression under at least one g* condition. From the approximate 44 k probe set list included in the Agilent microarrays, 2470 probe set have been significantly induced or repressed in at least one condition using a limma p value < 0.01 by FIESTA viewer v.1.0 (EXCEL format). Additional p values (limmaFDR, Rankprod & RankprodFDR) are also shown. Bars with a red star indicate that gene variation have statistical meaning. Genes that show no variations through our samples have been removed for convenience although the whole data is available at the *Gene Expression Omnibus (GEO) *database with the accession number [GEO:GSE29787]. Click here for file

Additional file 3 **Table S2. List of common probe sets representing up- or down-regulated genes in more than one experimental condition (EXCEL format)**. Fold change expression levels are indicated using three statistical confidence levels. Click here for file

Additional file 4 **Compressed GEDI analysis files have been uploaded as zip file, including each cluster list of probe sets and their expression ratio for each condition**. Click here for file
