# Supplementary material for: Gravitational and magnetic field variations synergize to cause subtle variations in the global transcriptional state of Arabidopsis in vitro callus cultures
Source: BMC Genomics. 2012 Mar 21;13:105. doi: 10.1186/1471-2164-13-105 (PMC3368779; doi:10.1186/1471-2164-13-105)
Supplement: Additional file 1 — Supplementary online material including additional figures (S1 & S2) and Table S1 has been uploaded as a pdf file. [file 1471-2164-13-105-S1.PDF]

Additional file 1. Supporting Information Online (Manzano et al. BMC genomics 2011)

FIGURE S1. Experiment set up in three Ground Based Facilities for altered gravity environment simulation
A) On the left photos of mechanical simulators of microgravity (µ*g*, RPM, real random mode) and hypergravity (2*g*, LDC) in DESC/ ESTEC (Noordwijk, The Netherlands) are shown. On the right the water-cooled duplex-Bitter magnet located at HFML with our samples placed inside (not visible). The temperature is controlled by a double-walled metal tube connected to a 22ºC water bath. A PVC spacer is used to place the stack of samples in the correct position. The stack of samples are contained in 40.8mm high tubes placed on top of each other at five effective g* levels. The spacing between the samples was 40.8 mm (as indicated in the right photo).
B) Picture of a 90 mm Petry dish containing the cell culture callus of Arabidopsis just before use in the RPM/LDC experiments (conveniently covered with aluminium foil to keep the sample in dark conditions).
C) Picture of the 5 tube collection before preparing the set up of one of three runs of the magnet experiment.

A)

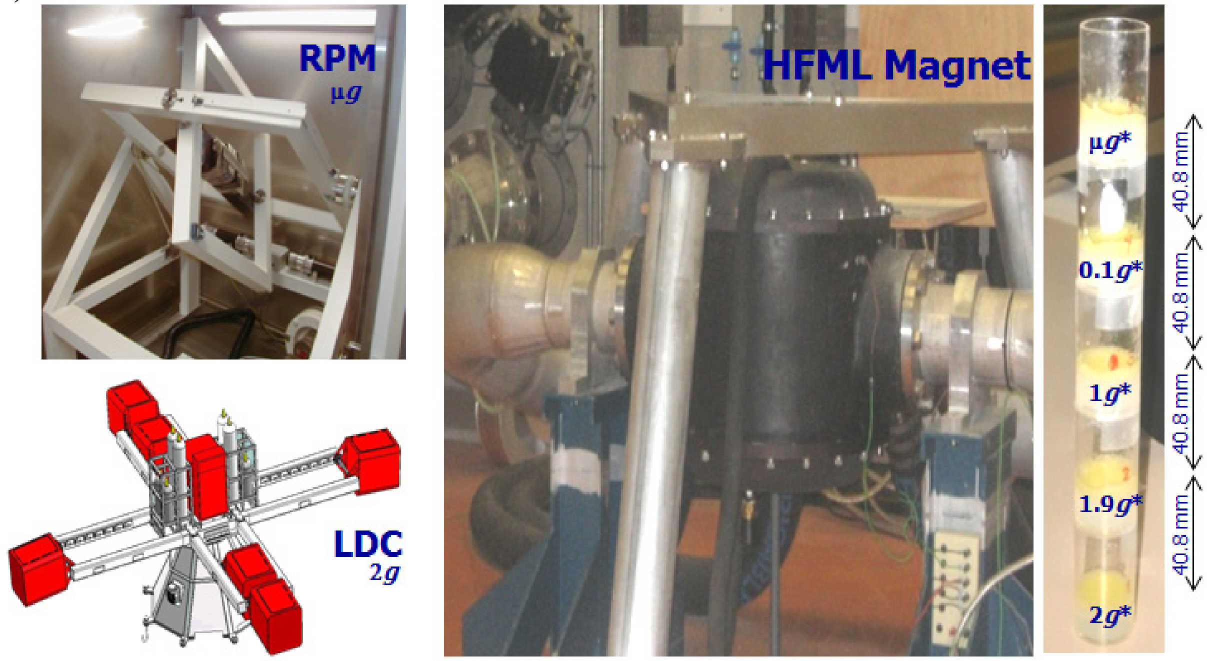

B)

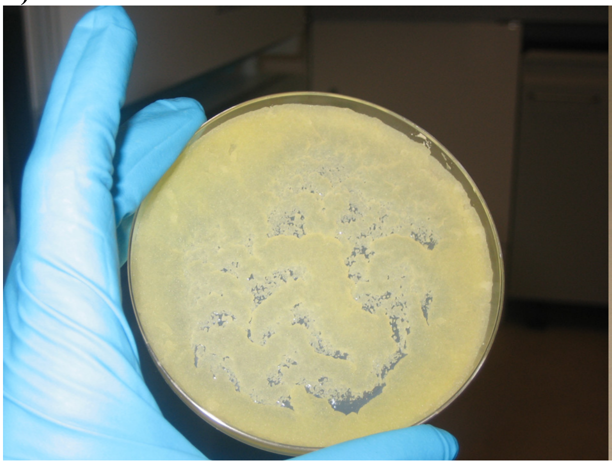

C)

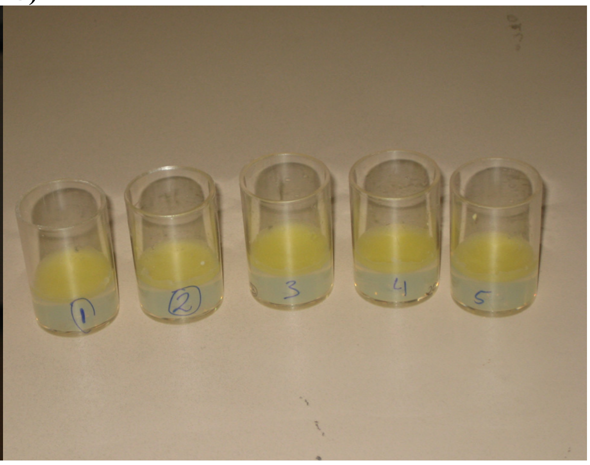

FIGURE S2. Specific probesets signal ratio determined by Agilent Arrays and by qPCR. Examples including down-regulated (left) and up-regulated (right) genes used for validation of the microarray technique. * indicates statistically different results versus 1g control. Notice that microarray analysis standard errors reached higher values than the qPCR results. Despite that, the same trend is observed using both techniques, so microarrays use is validated, although more precise (shorter error bars) and significant results are obtained by qPCR as expected.

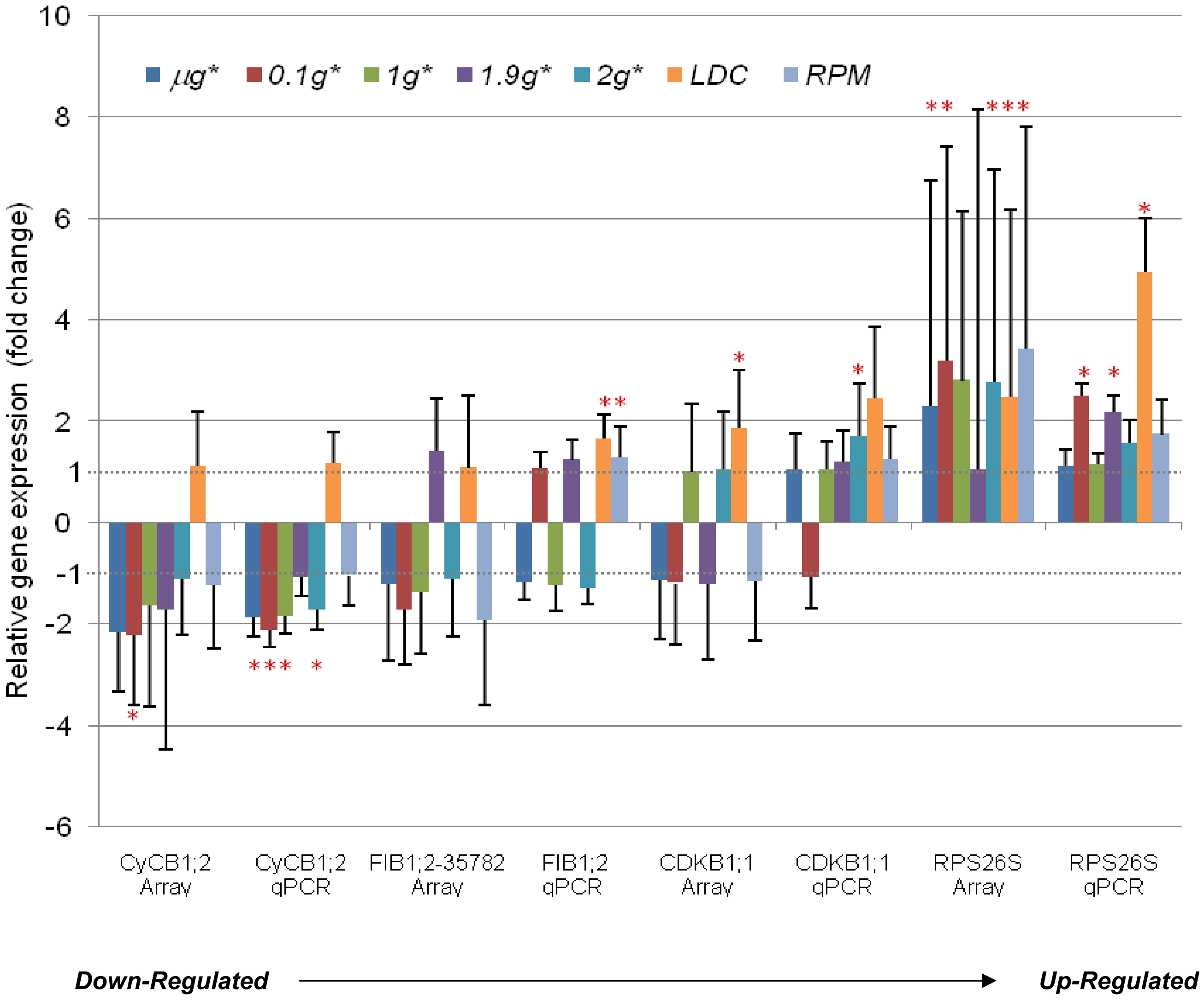

Table S3. Analysis of enriched ontology groups in genes showing opposite patterns in microgravity (RPM) and hypergravity (1.9g*) GEDI panels. The following GO groups are overrepresented as determined using BINGO 2.3. + indicates induced (Benjamini-Hodgberg False Discovery Rate corrected p-value <0.001, ++ if p-value <0.0005) and - indicates repressed genes.

Gene Ontology group	RPM & 1.9g (red areas)	RPM & 1.9g (blue areas)
amino acid and derivative metabolic process		--
binding		--
biosynthetic process	+	--
carbohydrate metabolic process		-
catabolic process		--
catalytic activity		--
cell	+	--
cell wall		--
cellular component organization and biogenesis		-
cellular process	+	--
chromatin binding	+	
cytoplasm	++	--
cytoskeleton		-
cytosol	+	--
DNA metabolic process	+	
embryonic development		-
endoplasmic reticulum	+	--
endosome		-
external encapsulating structure		--
extracellular region		--
generation of precursor metabolites and energy		--
Golgi apparatus		-
intracellular	++	--
kinase activity		-
membrane	+	--
metabolic process		--
mitochondrion		--
molecular_function		-
multicellular organismal development		--
nucleic acid binding		--
nucleobase, nucleoside, nucleotide and nucleic acid metabolic process	+	--
nucleolus		--
nucleoplasm		-
nucleotide binding		--
nucleus	+	--
peroxisome		-
photosynthesis	+	-
plasma membrane	+	--
plastid	++	--
post-embryonic development		-
protein binding		-
protein metabolic process		--
protein modification process		-
reproduction		-
response to abiotic stimulus	+	--
response to biotic stimulus		--
response to endogenous stimulus		-
response to stress		--
ribosome	++	--
RNA binding		--
structural molecule activity	++	--
thylakoid	+	--
transferase activity		-
translation	++	--
translation factor activity, nucleic acid binding		--
translation regulator activity		--
transport		-
tropism	+	
vacuole		--
